# 

**Published:** 1909-11

**Authors:** 


					﻿Sixty-fifth Year.
Vol. LXV. NOVEMBER, 1909.	No. 4
BUFFALO 
MEDICAL JOURNAL
AUSTIN FLINT M. D.	i
NOV.
EDITOR:
WILLIAM WARREN POTTER, M. D,
Vs G O /
ASSISTANT EDITORS:
WILLIAM C. KRAUSS, M. D.	NELSON W. WILSON, M. D.
ASSOCIATE EDITORS:
JAMES WRIGHT PUTNAM, M. D. ERNEST WENDE, M.D.
JOHN PARMENTER, M. D.	JOHN A. MILLER, Ph. D.
MAUD J. FRYE, M. D.
				

